# Activation and Connectivity within the Default Mode Network Contribute Independently to Future-Oriented Thought

**DOI:** 10.1038/srep21001

**Published:** 2016-02-12

**Authors:** Xiaoxiao Xu, Hong Yuan, Xu Lei

**Affiliations:** 1 Sleep and Neuroimaging Center, Faculty of Psychology, Southwest University, Chongqing, China; 2 Key Laboratory of Cognition and Personality of Ministry of Education, Chongqing, China

## Abstract

Future-oriented thought, a projection of the self into the future to pre-experience an event, has been linked to default mode network (DMN). Previous studies showed that the DMN was generally divided into two subsystems: anterior part (aDMN) and posterior part (pDMN). The former is mostly related to self-referential mental thought and latter engages in episodic memory retrieval and scene construction. However, functional contribution of these two subsystems and functional connectivity between them during future-oriented thought has rarely been reported. Here, we investigated these issues by using an experimental paradigm that allowed prospective, episodic decisions concerning one’s future (Future Self) to be compared with self-referential decisions about one’s immediate present state (Present Self). Additionally, two parallel control conditions that relied on non-personal semantic knowledge (Future Non-Self Control and Present Non-Self Control) were conducted. Our results revealed that the aDMN was preferentially activated when participants reflected on their present states, whereas the pDMN exhibited preferentially activation when participants reflected on their personal future. Intriguingly, significantly decreased aDMN-pDMN connectivity was observed when thinking about their future relative to other conditions. These results support the notion that activation within these subsystems and connectivity between them contribute differently to future-oriented thought.

A fundamental aspect of human consciousness relates to the ability to temporarily withdraw attention from the immediate environment to mentally simulate episodes that might happen in the future^1^. People engage in future-oriented thought with an astoundingly high frequency in daily life, which serves a number of important functions, including facilitating various kinds of goal-directed behaviors, supporting farsighted decision making and contributing to psychological well-being^2^,^3^. Excessively negative and unreasonable future thinking may lead to anxiety, adjustment disorder and even suicide. Some research indicated that depression, autism, schizophrenia and other diseases exhibited abnormal future thinking^4^,^5^,^6^. Due to its contribution to many important aspects of human cognition and behavior and the crucial role in various diseases, future-oriented thought has become the focus of growing interest in psychology and neuroscience in the last decade.

Recent functional magnetic resonance imaging (fMRI) studies have highlighted that a specific network of brain regions engaged in future-oriented thought. This network, referred to the default mode network (DMN), consists of the medial prefrontal cortex (mPFC), posterior cingulate cortex (PCC)/precuneus (PCu), inferior parietal lobe, lateral temporal cortex and hippocampal formation^7^. The DMN is typically deactivated during tasks requiring externally-oriented attention^8^,^9^ and activated during passive rest states or internally-oriented mental processes, such as autobiographical memory, theory of mind, self-referential processing and future thinking^10^,^11^. Future-oriented thought recruits multiple cognitive processes, including self-referential cognition^12^, a subjective sense of time^13^ and scene construction (i.e., the retrieval and integration of elements of previous experiences into a coherent event)^14^,^15^, which are served by a widely distributed set of brain regions within the DMN. Compared with imagining non-personal future events, imagining personal future events elicited stronger activation in the ventral mPFC and PCC^16^. Hassabis *et al.*^17^ showed that the hippocampus, parahippocampal gyrus, retrosplenial cortex and posterior parietal cortices were involved in the process of scene construction relative to the control task. D’Argembeau *et al.*^18^ revealed that the mPFC showed higher activation when reflecting on the immediate present self, whereas activation in right inferior parietal cortex was higher when reflecting on the future self. These evidences suggested that the DMN likely comprised multiple interacting subsystems.

An extensive body of literature about independent component analysis (ICA) has indicated that the DMN was generally divided into two subsystems: anterior part (aDMN) and posterior part (pDMN)^19^,^20^,^21^. The aDMN contains the mPFC, dorsal medial prefrontal cortex (dmPFC), anterior cingulate cortex, PCC, anterior temporal lobe, inferior frontal gyrus and lateral parietal cortex, whereas the pDMN consists of PCC, PCu, posterior inferior parietal lobule, angular, hippocampal and temporal lobe^19^,^20^,^21^. The mPFC and PCC are the hub regions of these two subsystems respectively. In a previous study, D’Argembeau *et al.*^18^ asked college undergraduates to perform four reflective tasks (reflecting on the present self, past self, present other and past other) and results showed that the mPFC were more recruited when reflecting on the present self than reflecting on the past self or reflecting on the other person. Another study suggested that the mPFC showed higher activation when reflecting on the present self than when reflecting on future and past selves^22^. Similarly, Ersner-Hershfield *et al.*^23^ found that rostral anterior cingulate cortex, a region of the aDMN, was more activated in the present self than in the future self. Compared to the present self, activation in right inferior parietal cortex, a node of the pDMN, was higher when reflecting on the future self^18^. Moreover, the posterior inferior parietal lobule, retrosplenial cortex, parahippocampal gyrus and hippocampal formation were more sensitive to the act of simulating the future using mnemonic imagery-based processes^11^. Taken together, these evidences indicated that some regions belonging to the aDMN showed higher activation when reflecting on their present and other nodes which belong to the pDMN were preferentially activated when reflecting on their future. However, these seed-based studies do not clarify whether other brain regions are involved in events of interest. In contrast to this approach, ICA could elucidate extensive brain networks subserving future thinking. More interestingly, though numbers of ICA-based studies have indicated that the DMN consists of two components (the aDMN and the pDMN), our knowledge of their contribution to future-oriented thought is still limited.

In modern neuroscience, the complex brain is considered to be an effective and network, and numbers of different brain regions implement and perform diverse tasks and functions. They are not isolated, but constant to exchange and share neural information. Functional connectivity is defined as the temporal dependence of neuronal activity patterns of anatomically separated brain regions, reflecting the level of functional communication between regions. Previous study has emphasized the resting-state functional connectivity between subsystems within the DMN by seed-based method^11^, and its essence is connection between these seed regions. Contrast to this connectivity, functional connectivity between independent components which are obtained by a data driven method of ICA reflects large scale brain networks connections. In this study, we are concerned about the functional connectivity between the aDMN and the pDMN components during future thinking. If these two subsystems within the DMN are engaged in different subfunctions during future-oriented thought, some changes in the aDMN-pDMN connectivity will be expected.

Here, we aimed to investigate the functional specialization and functional connectivity within the subsystems of the DMN during future-oriented thought. A recent study showed that activation and functional connectivity within the DMN contributed differently to externally-oriented process^24^, and we were interested in the contribution of activation and functional connectivity within these two subsystems to internally-oriented process. To this aim, our participants were asked to make prospective, episodic decisions about themselves (Future Self) and self-referential decisions regarding their immediate mental state or present situation (Present Self). Two parallel control conditions (Future Non-Self Control/Future Ctrl and Present Non-Self Control/Present Ctrl) were also conducted. An example of a single trial is shown in Fig. 1. Following the future-oriented thought task with fMRI scans, the series of questions were presented again and subjects were asked about the strategies used to answer each question. For the fMRI data, two functional networks: the aDMN and the pDMN were extracted by the method of ICA, and correlation coefficients for each task condition of each subject were introduced into a one-way analysis of variance (ANOVA) to reveal the aDMN-pDMN connectivity during future thinking.

## Results

### Reaction time

During the fMRI scanning, participants had to perform 72 items and 18 items for each condition (Future Self, Present Self, Future Ctrl and Present Ctrl). Each item comprised a context setting statement and a question. They were given 10 s to read the contextually orienting sentence and choose their answer with a key press. Task conditions varied with respect to reaction time (mean ± standard error: Future Self = 6224 ± 242 ms; Present Self = 6097 ± 224 ms; Future Ctrl = 6373 ± 258 ms; Present Ctrl = 6310 ± 245 ms). A one-way repeated-measures ANOVA revealed significant differences (*F* (3, 87) = 3.32, *p* = 0.023) among the four conditions. Paired *t*-tests further revealed that the reaction time in Present Self was significantly shorter than in Future Ctrl (t_29_ = −2.88, *p* = 0.007) and in Present Ctrl (t_29_ = −2.25, *p* = 0.03), but there was no difference between Present Self and Future Self (t_29_ = 1.48, *p* = 0.15). And Future Self, Future Ctrl and Present Ctrl did not differ from each other (t_29_ < −0.61, *p* > 0.12). In addition, compared with the speed of their responses to the non-self control condition (Future Ctrl and Present Ctrl), participants responded faster to the self condition (Future Self and Present Self) (self = 6161 ± 229 ms; non-self control = 6342 ± 246 ms) (t_29_ = −2.89, *p* = 0.007), but there was no significantly different between the future condition (Future Self and Future Ctrl) and the present condition (Present Self and Present Ctrl) (future = 6298 ± 246 ms; present = 6204 ± 230 ms) (t_29_ = 1.47, *p* = 0.15). These results were consistent with previous research findings^11^, demonstrating the faster response when reflecting on self related information than non-self related information. Unless mentioned otherwise, all the *p* values reported in the current study were corrected by Bonferroni correction.

### Strategy probe questions

In order to confirm the experimental conditions differed as expected and probe the strategies during decision making, subjects were asked about strategies used to answer each question, including three factors of “mental-imagery”, “vividness” and “self-projection”. Strategy probe questions obtained following the fMRI scans confirmed that the four conditions differed as expected. We found significant differences of mental-imagery across conditions (mean ± standard error: Future Self = 4.86 ± 0.57; Present Self = 4.52 ± 0.76; Future Ctrl = 4.31 ± 0.80; Present Ctrl = 4.19 ± 0.78) (*F* (3, 87) = 14.86, *p* < 0.001) and paired *t*-tests further revealed that the highest score of mental-imagery was observed when participants reflected on Future Self than when reflecting on Present Self (t_29_ = 2.79, *p* = 0.009), Future Ctrl (t_29_ = 4.67, *p* < 0.001) and Present Ctrl (t_29_ = 5.47, *p* < 0.001) (Fig. 2A). Vividness rating also varied across conditions (Future Self = 5.07 ± 0.56; Present Self = 4.67 ± 0.67; Future Ctrl = 4.28 ± 0.82; Present Ctrl = 4.21 ± 0.81). One-way ANOVA showed significant differences (*F* (3, 87) = 24.33, *p* < 0.001) and paired *t*-tests further revealed the highest score of vividness during reflection about Future Self than when reflecting on Present Self (t_29_ = 3.81, *p* = 0.001), Future Ctrl (t_29_ = 5.89, *p* < 0.001) and Present Ctrl (t_29_ = 6.13, *p* < 0.001) (Fig. 2B). Large differences in participants’ sense of self-projection were observed (Future Self = 5.43 ± 0.80; Present Self = 4.80 ± 0.91; Future Ctrl = 3.36 ± 1.10; Present Ctrl = 3.26 ± 1.16) (*F* (3, 87) = 59.74, *p* < 0.001). After further paired *t*-tests, we found that participants engaged the most self-projection when reflecting on Future Self than when reflecting on Present Self (t_29_ = 4.74, *p* < 0.001), Future Ctrl (t_29_ = 8.44, *p* < 0.001) and Present Ctrl (t_29_ = 8.44, *p* < 0.001) (Fig. 2C). Besides, we also conducted correlation analysis and found that these three strategies were closely related to each other. More specifically, the mental-imagery was significantly correlated with vividness (r (30) = 0.95, *p* < 0.001) and self-projection (r (30) = 0.74, *p* < 0.001), and vividness was correlated with self-projection (r (30) = 0.80, *p* < 0.001).

Mental-imagery, vividness and self-projection are the pivotal indexes to measure future-oriented thought. The higher scores participants obtained, the more internally-oriented thought they engaged. Of note, Future Self experienced the highest levels of mental-imagery, vividness and self-projection, showing that reflecting on the future self may simultaneously engage a quantity of component processes served by a widely distributed set of brain regions.

### Functional network

By applying spatial ICA to task-state fMRI data, we found that two networks were largely overlapped with the DMN. The first network corresponded to the aDMN, which encompassed the dmPFC, medial frontal gyrus, inferior frontal gyrus, temporal parietal junction (TPJ), PCC and PCu. The second network overlapped more closely related to the pDMN and it consisted of the PCC, PCu, superior frontal gyrus, middle frontal gyrus, posterior inferior parietal lobule, middle temporal gyrus, angular gyrus, hippocampal formation and parahippocampal cortex. The spatial anatomy of two networks were presented in Fig. 3, and the anatomical locations and the corresponding Montreal Neurological Institute (MNI) template space^25^ coordinates of the brain regions were summarized in Table 1.

### Task-related functional network activation

We examined the mean magnitude of task-related hemodynamic response within each network (Fig. 4). A one-way ANOVA assessed signal change of the aDMN and activation significantly varied among these task conditions (*F* (3, 87) = 448.88, *p* < 0.001) and the aDMN showed highest activity when reflecting on Present Self than when reflecting on Future Self (t_29_ = 10.75, *p* < 0.001), Future Ctrl (t_29_ = 42.38, *p* < 0.001) and Present Ctrl (t_29_ = 24.63, *p* < 0.001) (Fig. 4A). Similarly, one-way ANOVA revealed significant differences of signal change within the pDMN (*F* (3, 87) = 925.84, *p* < 0.001) and activation in this brain region was highest when thinking about Future Self relative to Present Self (t_29_ = 26.12, *p* < 0.001), Future Ctrl (t_29_ = 31.22, *p* < 0.001) and Present Ctrl (t_29_ = 41.23, *p* < 0.001) (Fig. 4B). These results showed that the aDMN subsystem was preferentially activated in Present Self and the pDMN subsystem was preferentially activated in Future Self.

### Altered functional connectivity between the aDMN and the pDMN

After the functional connectivity analysis, we compared Pearson’s correlation between the aDMN and the pDMN across tasks conditions using the one-way ANOVA, testing for significant differences (*F* (3, 87) = 13.09, *p* < 0.001). And paired *t*-tests further revealed the weakest connectivity between the aDMN and the pDMN during reflection about Future Self than when reflecting on Present Self (t_29_ = –7.42, *p* < 0.001), Future Ctrl (t_29_ = –3.45, *p* = 0.002) and Present Ctrl (t_29_ = –4.33, *p* < 0.001) (Fig. 5).

## Discussion

In the current study, we mainly explored the functional contribution of the aDMN and the pDMN and their functional connectivity during future-oriented thought. There were three main findings. Firstly, compared with other three conditions, participants engaged the highest levels of mental-imagery, vividness and self-projection when reflecting on their future, which may suggest the predominant cognitive function when imagining the future self. Secondly, we found a functional dissociation within the DMN. To be specific, the aDMN was more activated when participants made decisions about their present, whereas the pDMN preferentially activated when participants made decisions about their future. Thirdly, a significant decrease of aDMN-pDMN connectivity was observed when thinking about Future Self with respect to Present Self, Future Ctrl and Present Ctrl. These results indicated that functional divisions of anterior-posterior subsystems of the DMN and different patterns of aDMN-pDMN functional connectivity contributed differently to future-oriented thought.

In this study, we found that the aDMN showed higher activation in Present Self than Future Self, Future Ctrl and Present Ctrl, which was in line with previous studies. For example, twenty-one college students were asked to reflect on the present self, past self, present other and past other^18^. Compared with reflecting on the past self or reflecting on the other person, a key node of the aDMN (i.e., mPFC) was more recruited when reflecting on the present self^18^. In another study, activation within the aDMN (including the mPFC) was higher when reflecting on the present self than the past and future selves^22^. The mPFC has been frequently associated with self-referential processing^26^, subjective appraisal of the personal relevance of mental contents^11^,^27^ and motivational impact of future thinking^28^. Except for the mPFC, the TPJ also engaged when reflecting on Present Self^11^. Some researchers declared that the representation of mental states was subserved by the TPJ. For example, Samson and colleagues proposed that the left TPJ had a vital role in representing mental states^29^ and Saxe suggested that the right TPJ was crucial for the representation of mental states, particularly false beliefs^30^. D’Argembeau *et al.*^31^ showed the vast majority of immediate present thoughts (or near-future thoughts) dealing with action planning and personal goals, which preferentially engaged the activity of the aDMN^28^. Hence, these evidences suggested that activation within the aDMN reflected one’s immediate present situation or mental states, especially the personal goal and action planning.

The pDMN was observed preferentially activated when participants thought about Future Self relative to other conditions. As illustrated in Table 1, the pDMN encompassed the PCC, PCu, superior frontal gyrus, middle frontal gyrus, posterior inferior parietal lobule, middle temporal gyrus, angular gyrus, hippocampal formation and parahippocampal cortex, which were activated in episodic/contextual retrieval^32^ and simulating one’s future^15^. For instance, previous task-related neuroimaging studies have indicated that the PCC/PCu increases activation in successful retrieval of episodic memory^33^ and visuo-spatial imagery^34^. Future thinking needs to retrieve episodic memory and flexibly recombine these pieces of information into a coherent mental scene, and activation in the temporal lobe area was hypothesized to play a pivotal role in mental scene construction^14^. The hippocampus has long been thought to be related to episodic memory and spatial memory^35^. Damage to the hippocampus often leads to deficits in imagining^36^, in spite of preserved narrative processing^37^. Lesions to the parahippocampal cortex can impair spatial and scene recognition^38^, while the angular gyrus damage lead to broad deficits in recollective aspects of episodic memory^39^. The right inferior parietal cortex showed higher activity when reflecting on the future self than the present self^18^. In addition, compared with the personal goal and mind wandering, the pDMN (including PCC, posterior inferior parietal lobule, hippocampus and parahippocampal) showed stronger activation in episodic future thinking^28^. Combined with these studies, the increased activation in the pDMN during the future self was associated with simulating the future using mnemonic imagery-based processes.

In summary, the DMN had a crucial role in future-oriented thought and subsystems engaged distinct subfunctions. As mentioned above, substantial evidence showed that the whole DMN was usually divided into several subsystems, but there was still little known about their contributions to future thinking. Andrews-Hanna *et al.*^11^ used both resting state recording and experimental manipulations to provide evidence for the separation of the DMN. They found two subsystems within the DMN, linked by a “midline core”. The first subsystem termed the “dmPFC subsystem”, comprising of the dmPFC, lateral temporal cortex, TPJ and temporal pole, showed preferentially activation when participants reflected on their present mental states. The second subsystem termed the “medial temporal lobe (MTL) subsystem”, including the ventral mPFC, hippocampal formation, parahippocampal cortex, retrosplenial cortex and posterior inferior parietal lobule, was associated with memory-based scene construction during episodic future thinking. Both subsystems were tightly connected to “hub” regions including anterior mPFC and PCC, activating preferentially for self-relevant conditions. However, this model driven method couldn’t identify additional regions with extended activation in response to future-oriented thought. The method of ICA could elucidate large scale brain networks subserving future thinking and it was applied here to investigate contributions of the aDMN and the pDMN to future-oriented thought. In contrast to the work of Andrews-Hanna *et al.*^11^, though there were some similarities about the anatomy and functions between the aDMN/pDMN subsystems and the dmPFC/vmPFC subsystems, the former contained more brain regions than the latter. Most importantly, we explored the functional connectivity between subsystems when participants reflected on future-oriented thought, which was rarely mentioned in previous studies. Another difference with Andrews-Hanna *et al.*^11^ was resting-state functional connectivity between subsystems and its essence of this connectivity was connection between seed regions. Compared with this approach, functional connectivity in our study reflected large scale brain networks connections, i.e., the correlation coefficients between independent components.

To the best of our knowledge, this study is the first to explore functional connectivity between subsystems within the DMN during future-oriented thought. We found a significantly decreased connectivity between the aDMN and the pDMN when thinking about Future Self relative to Present Self, Future Ctrl and Present Ctrl, without difference among these three conditions. Combined with our behavioral results that Future Self experienced the highest levels internally-oriented processes, we considered that the decreased aDMN-pDMN connectivity may be relevant to internally-oriented processes and the increased aDMN-pDMN connectivity was likely related to cognitive control factors or externally-oriented processes. Previous literature approved our suppositions, in which the increased connectivity within the DMN facilitated or monitored cognitive performance^24^, whereas the decreased connectivity reflected declined present-moment awareness and hindered cognitive performance^40^,^41^,^42^. For example, the decreased connectivity within the DMN has been shown to underlie deficits in attention control and externally directed cognitive process (i.e., working memory)^43^,^44^. Moreover, some researchers indicated that the decreased connectivity within the DMN was connected to internally-oriented cognition, such as self-related thought and vivid future imagination. For instance, in a self-referential processing task, researchers found that coupling between subregions of the DMN was reduced during the self condition compared to control condition^45^. Østby *et al.*^46^ explored both resting-state functional and structural brain correlates of vividness scores in future imagination in children and adolescents. And the results showed that the reduced connectivity within the DMN was related to higher vividness of future episodes.

Furthermore, it is interesting to compare the aDMN-pDMN functional connectivity between Future Self and Present Self, a wide difference in functional connectivity for the temporal distance. Previous study had suggested that functions of future-oriented thought differed according to temporal distance, with the vast majority of immediate present thoughts (or near-future thoughts) dealing with action planning, whereas future thoughts were more evenly distributed across various functions (i.e., decision making, emotion regulation and action planning)^31^. In addition, there is evidence that people represent the immediate present (or near future) events more in terms of concrete details about the means for achieving their goals (e.g., the “how” details of the action), whereas the far future events more in terms of abstract goal-related knowledge (e.g., the “why” aspects of an action)^47^. Another discrepancy between Future Self and Present Self is that some events have been happened in details and are anticipated with high degrees of certainty during the present processing, whereas the future processing is much more open and uncertain^1^. In our study, participants had to make decisions about their current situation and personal future. According to above studies, Present Self is more certain and has a definite answer. Thinking about Present Self involves the concrete details of action planning or the process of goal attainment, which contains more externally-directed processes. Comparatively, the future is more open, uncertain and relates to the abstract goal-related knowledge. It’s possibly hard to make a reasonable decision and participants have to conduct more internally-oriented mental thought during Future Self. Therefore, an increased connectivity within the DMN was observed when participants reflected on Present Self, whereas a decreased connectivity was found when participants reflected on Future Self.

Besides, it’s worth noting that the decreased aDMN-pDMN connectivity when reflecting on the personal future is distinctly different from other experimental conditions, such as sleep deprivation^48^, aging adults^49^, Alzheimer’s patients^50^, patients with Autism spectrum disorder^51^ and Attention-deficit hyperactivity disorder^43^. The decreased connectivity during future-oriented thought has a clear adaptive value, enabling one act flexibly in the present to increase future chances of survival. However, the decreased connectivity during sleep deprivation, aging adults and disease disorders indicates the deficit of cognitive processing or dysfunction^52^. For example, the decreased resting state functional connectivity between the aDMN and the pDMN suggests an absence of self-referential thought in autism^53^. Overall, the functional connectivity within the DMN is related to the specific task conditions and our finding provides an alternative perspective to understand the neural mechanism of future-oriented thought.

In conclusion, we reinforced the notion that subsystems of the DMN were involved in different functions and this study was the first to explore the aDMN-pDMN functional connectivity during future-oriented thought. Our results revealed that the aDMN was more activated when participants reflected on their present mental state and the pDMN preferentially activated when participants imagined future scenarios about themselves. Furthermore, functional connectivity analysis revealed a significantly decreased connectivity between the aDMN and the pDMN during Future Self relative to other conditions. These results indicated that activity and functional connectivity within the DMN contribute differently to future-oriented thought.

## Materials and Methods

### Participants

Thirty healthy right-handed subjects participated in the present study, age-range between 18 and 23 years (mean = 20.3, SD = 1.3, female = 13). All participants were recruited from the local community through advertisements. They had no history of psychiatric or neurological illness as confirmed by psychiatric clinical assessment. Written informed consent was obtained after detailed explanation of the study protocol. The study was approved by the Ethics Committee of Southwest University, and all procedures involved were in accordance with the sixth revision of the Declaration of Helsinki.

### Experimental design

Participants performed a future-oriented thought task with fMRI scans. More specifically, they were asked to make prospective, episodic decisions about themselves (Future Self), decisions regarding their immediate present situation or mental state (Present Self) and two parallel control conditions (Future Ctrl and Present Ctrl). This word-cuing paradigms or contextually-orienting sentences are widely used in experiments about imagining the future, which are proved to be effective in producing internally-oriented thought^1^,^11^. Most of the materials are self-related or familiar to individual and they can easily trigger mental imagery process. Furthermore, with inducing the retrieval and integration of relevant elements of previous experiences (which requires attention to internal mental processes), participants mentally generate and maintain a complex and coherent scene or event which greatly reduced the influence of external stimulation on internal mental processes. Finally, the matching of sentence structure, word number and reading time across conditions also reduces effects of external attention on future thinking and persists the differentiation of internally-oriented thought.

### Procedure

**Figure 1:**
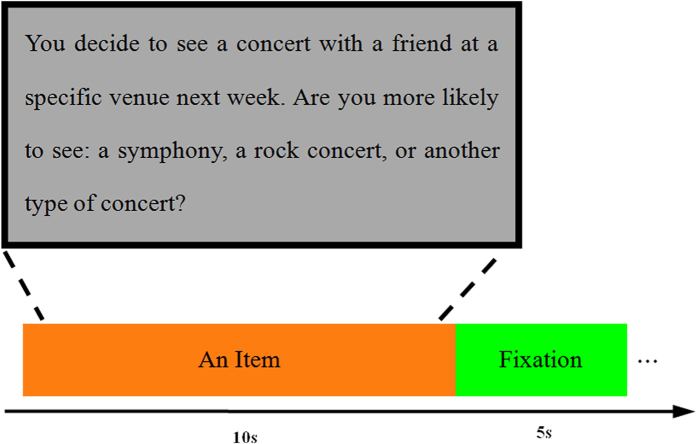
An example procedure for a single trial of future-oriented thought. Each item comprised a context setting statement and a question. Participants were given 10 s to read the contextually orienting sentence, imagine the event and choose their answer with a key press from three possible alternative answers, and 5 s of fixation separated items.

**Figure 2:**
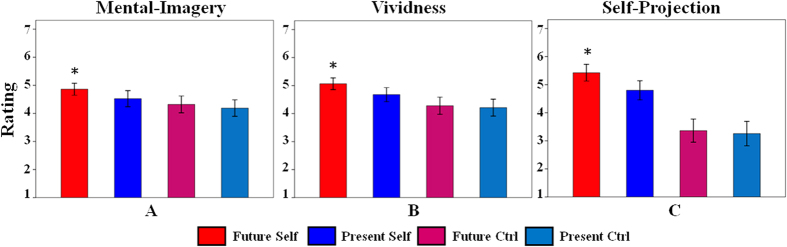
Scores of strategy probe questions across task conditions. One-way repeated-measures ANOVA (*p* < 0.001) revealed the significant differences of mental-imagery, vividness and self-projection across task conditions, respectively. And multiple comparisons (paired *t*-tests) found that Future Self experienced the highest levels of mental-imagery (**A**), vividness (**B**) and self-projection (**C**) relative to Present Self, Future Ctrl and Present Ctrl. *indicates significant differences at *p* < 0.01.

**Figure 3:**
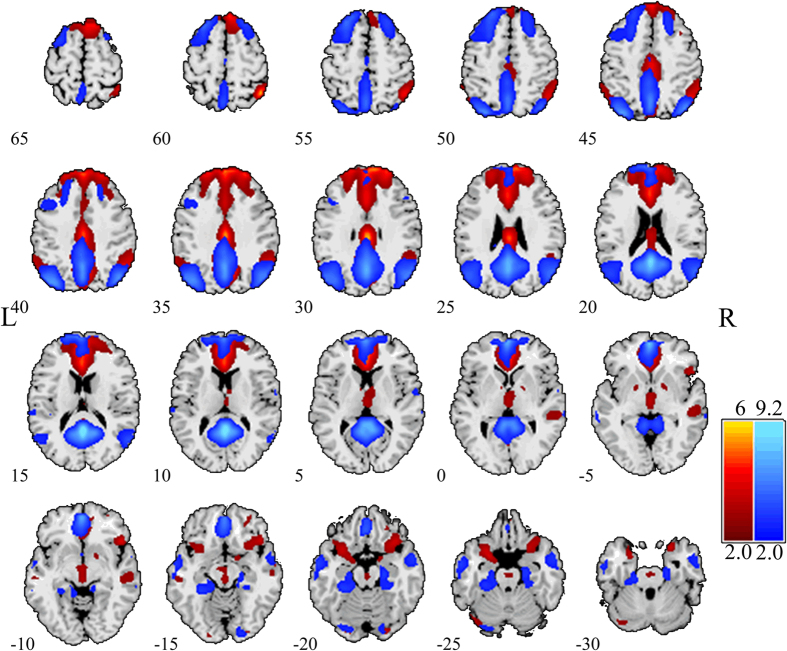
The spatial distribution of the aDMN (red) and the pDMN (blue). Brain areas with intensities of two standard deviations greater than the mean are shown. aDMN, anterior default mode network; pDMN, posterior default mode network.

**Figure 4:**
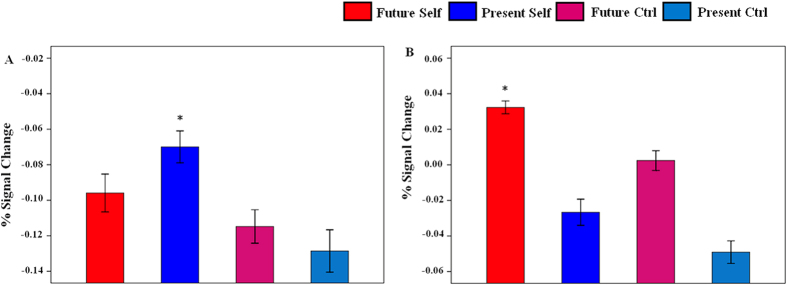
Mean and standard error of task-related percent BOLD signal change within each network. (**A**) The aDMN subsystem was preferentially activated when participants reflected on their present or mental states. (**B**) In contrast, the pDMN subsystem exhibited preferentially activation when participants reflected on their personal future. Error bars represent standard error of the mean. *indicates significant differences at *p* < 0.01.

**Figure 5:**
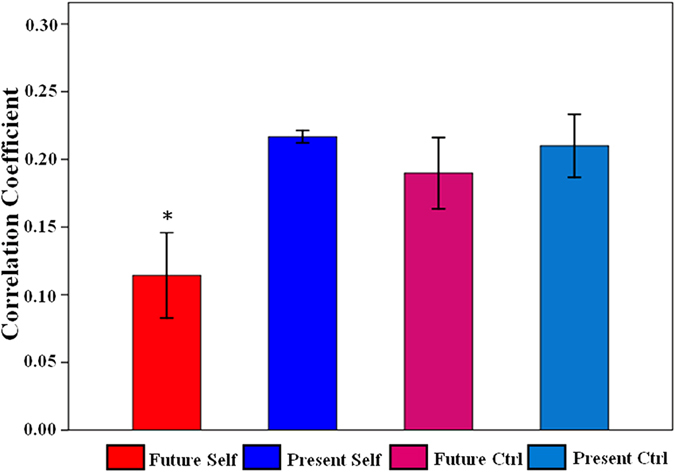
Functional network connectivity between the aDMN and the pDMN altered across task conditions. Significant alteration across conditions was assessed by one-way repeated-measures ANOVA and the aDMN-pDMN connectivity significantly (paired *t*-tests, *p* < 0.01) decreased when participants reflected on Future Self relative to Present Self, Future Ctrl and Present Ctrl.

Before the fMRI scanning, participants had to learn how to perform this experiment by 8 items, 2 items for each condition. As illustrated in Fig. 1, each item comprised a context setting statement and a question. For example, “You decide to see a concert with a friend at a specific venue next week. Are you more likely to see: a symphony, a rock concert, or another type of concert”. Participants were given 10 s to read the contextually orienting sentence and imagine it as vivid as possible. This procedure required a detailed construction of the event, in particular, when and where the event occurred, the persons and objects that would be present, their actions and feelings. Then they also need choose their answer with a key press from three possible alternative selections and reaction time of making decision was used to assess whether they conducted mental imagery successfully. The 5 s of fixation separated items. During the fMRI scanning, participants performed the future-oriented thought task with 72 items according to the experimental instruction. In order to confirm the experimental conditions differed as expected and probe the strategies used to answer the series of 72 questions, subjects assessed each question from three aspects after the fMRI scanning, comprising “mental-imagery”, “vividness” and “self-projection”. These strategies used a Likert scale, where 1 represented “not at all” and 7 represented “a lot.”

### Image acquisition

High-resolution T1-weighted structural images were acquired using a 3 T Siemens Trio scanner. The 3D spoiled gradient recalled (SPGR) sequence used the following parameters: TR/TE = 8.5/3.4 ms, FOV = 240 × 240 mm^2^, flip angle = 12°, acquisition matrix = 512 × 512, thickness = 1 mm with no gap. The high-resolution T1-weighted structural images provided an anatomical reference for the functional scans. Subsequently, 364 fMRI volumes were acquired using an EPI sequence with the following parameters: TR = 1500 ms, TE = 29 ms, flip angle = 90°, acquisition matrix = 64 × 64, in-plane resolution = 3.0 × 3.0 mm^2^, FOV = 192 × 192 mm^2^, axial slices = 25, thickness/gap = 5/0.5 mm. Head movements were minimized by using a cushioned head fixation device.

### Preprocessing and functional network definition

All the fMRI data were mainly preprocessed with the SPM8 (http://www.fil.ion.ucl.ac.uk/spm/, Welcome Department of Cognitive Neurology, UK). The preprocessing steps included slice timing, head motion correction, spatial normalization, smoothing (6-mm full width at half maximum Gaussian kernel). After fMRI data preprocessing, we performed group ICA using the GIFT toolbox (http://icatb.sourceforge.net/)^54^ to retrieve brain networks of interest. The optimal number of components was set to 25, which was estimated using the minimum description length criterion^55^. Previous fMRI studies using ICA suggested that 25 independent components can provide a reliable representation of large-scale networks^56^. After data reduction by principal component analysis, ICA decomposition was performed on concatenated datasets using the Extended Infomax algorithm. Independent components and time courses for each subject were back-reconstructed, and the spatial maps for each subject were entered into a one-*sample t*-test to identify voxels with activities that were significantly different from zero. The threshold for significance was set using a family wise error correction (FWE procedure, *p* < 0.05). We employed the DMN maps from one of our previous resting state fMRI studies as spatial templates for component classification^21^. The selected networks corresponded to those components with the largest spatial correlations with the templates and with correlation values at least double that of all other networks. Finally, two components of interest were chosen for further analysis, including the aDMN and the pDMN. The time courses of the selected components were used as input for correlation analyses.

### Activation and functional connectivity analysis

In activation analysis, we assessed the mean task-related hemodynamic response within the aDMN and the pDMN. In functional connectivity analysis, the time courses of the selected components were used as inputs for correlation calculations. We used the functional network connectivity toolbox^57^ to calculate the connectivity. The correlation coefficients were normalized to z-scores with Fisher’s r-to-z transformation in order to increase the normality of the distribution, allowing further statistical analysis of correlation strengths.

### Statistical analysis

For the behavioral data, one-way ANOVA revealed that conditions varied significantly with respect to reaction time. For the strategy probe questions, including mental-imagery, vividness and self-projection, one-way ANOVA and paired *t*-tests were used to access the differences among task conditions.

For the fMRI data, we performed one-way ANOVA to determine significant differences in percent BOLD signal changes of the aDMN and the pDMN across task conditions, respectively. Pearson correlations between time courses of the aDMN and the pDMN components were calculated for between-network (aDMN-pDMN) functional connectivity for each task condition. Correlation coefficients for each task condition of each subject were also introduced into the one-way ANOVA.

## Additional Information

**How to cite this article**: Xu, X. *et al.* Activation and Connectivity within the Default Mode Network Contribute Independently to Future-Oriented Thought. *Sci. Rep.* **6**, 21001; doi: 10.1038/srep21001 (2016).

## Figures and Tables

**Table 1:** Peak foci for the aDMN and the pDMN defined by group ICA.

Regions	MNI coordinates			*T*
	X	Y	Z	
aDMN				
L Dorsal medial prefrontal cortex	−6	57	27	7.42
R Dorsal medial prefrontal cortex	12	48	33	7.19
R Medial frontal gyrus	12	30	69	5.60
Cingulum_Mid	0	−12	36	7.18
L Inferior frontal gyrus	−30	18	−21	5.94
R Temporal parietal junction	60	−48	33	5.67
L Precuneus	−6	−66	27	5.66
R Precuneus	6	−66	39	6.35
Posterior cingulate cortex	−6	−45	24	5.85
pDMN				
Posterior cingulate cortex	6	−54	18	11.39
L Precuneus	−3	−63	33	11.05
R Precuneus	6	−63	36	10.48
L Precuneus	−39	−72	33	9.71
L Superior frontal gyrus	−12	45	45	8.62
R Superior frontal gyrus	27	30	54	10.25
L Middle frontal gyrus	−30	24	42	8.85
L Posterior inferior parietal lobule	−57	−66	21	9.59
R Posterior inferior parietal lobule	48	−60	27	10.05
L Middle temporal gyrus	−45	−69	21	9.59
R Middle temporal gyrus	45	−66	21	8.98
R Angular	42	−78	42	8.05
R Hippocampal formation	21	−18	−24	7.24
R Parahippocampal gyrus	30	−27	−18	6.68

Abbreviations used: aDMN, anterior default mode network; pDMN, posterior default mode network; L, left hemisphere; R, right hemisphere. The significance threshold was set to *p* < 0.05, FWE-corrected.
